# Molecular phenotypes of colorectal cancer and potential clinical applications

**DOI:** 10.1093/gastro/gov046

**Published:** 2015-09-03

**Authors:** Jonathan M. Kocarnik, Stacey Shiovitz, Amanda I. Phipps

**Affiliations:** ^1^Public Health Sciences Division, Fred Hutchinson Cancer Research Center, Seattle, WA, USA,; ^2^Epidemiology Department, University of Washington, Seattle, WA, USA,; ^3^Clinical Research Division, Fred Hutchinson Cancer Research Center, Seattle, WA, USA and; ^4^Department of Medicine, Division of Medical Oncology, University of Washington, Seattle, WA, USA

**Keywords:** colorectal cancer, microsatellite instability, CpG island methylator phenotype, *KRAS*, *BRAF*, treatment, epidemiology

## Abstract

Colorectal cancer (CRC) is a heterogeneous disease, arising from many possible etiological pathways. This heterogeneity can have important implications for CRC prognosis and clinical management. Epidemiological studies of CRC risk and prognosis—as well as clinical trials for the treatment of CRC—must therefore be sensitive to the molecular phenotype of colorectal tumors in patients under study. In this review, we describe four tumor markers that have been widely studied as reflections of CRC heterogeneity: (i) microsatellite instability (MSI) or DNA mismatch repair (MMR) deficiency, (ii) the CpG island methylator phenotype (CIMP), and somatic mutations in (iii) *BRAF* and (iv) *KRAS.* These tumor markers have been used to better characterize CRC epidemiology and, increasingly, may be used to guide clinical decision-making. Going beyond these traditional tumor markers, we also briefly review some more novel markers likely to be of clinical significance. Lastly, recognizing that none of these individual tumor markers are isolated attributes but, rather, a reflection of broader tumor phenotypes, we review some of the hypothesized etiological pathways of CRC development and their associated clinical differences.

## Introduction

As with most forms of cancer, colorectal cancer (CRC) is a biologically and epidemiologically heterogeneous disease. Such heterogeneity reflects the fact that there are many possible etiological pathways responsible for driving CRC development, each of which may be marked by distinct driver mutations and genetic or epigenetic signatures. Importantly, this heterogeneity can also have implications for CRC prognosis and the clinical management of this disease. Efforts to characterize molecular phenotypes and subtype classifications for CRC thus hold relevance across the spectrum of the disease’s natural history—from understanding how CRC develops and who is at risk, to guiding treatment decisions and secondary prevention in an informed manner.

Until recently, studies accounting for possible heterogeneity in the epidemiology and etiology of CRC have been limited to the consideration of higher-level tumor attributes, such as tumor site (e.g. colon or rectum). For example, previous studies have suggested that certain lifestyle factors, such as cigarette smoking, are more strongly associated with risk of rectal cancer than with risk of colon cancer [1, 2]; however, in light of increasing evidence indicating that the molecular profile of CRC differs greatly according to tumor site [3, 4], more sensitive epidemiological studies exploring possible etiological differences across specific molecular phenotypes of disease need to be conducted.

Similarly, with respect to clinical management, the use of surgery, chemotherapy and/or radiation therapy for CRC has long been guided by the TNM stage classification and tumor site [5], without consideration of molecular attributes. Stage I (T1–2 N0) colon or rectal cancer is treated with surgery or endoscopic removal of the tumor alone. Patients with Stage II–III (T3–4 N0, Tx N1–2) rectal cancer receive, as standard, neoadjuvant chemoradiation with either 5-fluorouracil (5-FU) or oral capecitabine [6, 7]. The current standard of care for Stage III (Tx N1–2) colon cancer is adjuvant therapy, i.e. six months of 5-fluorouracil (5-FU) and oxaliplatin (FOLFOX) chemotherapy [8]. Although there is a clear benefit from adjuvant treatment in the setting of Stage III colon cancer, approximately one Stage III patient in three will still experience recurrence within five years [9]; the utility of adjuvant chemotherapy in Stage II (T3–4 N0) colon cancer remains controversial, even when it is restricted to patients with high-risk clinical features [10, 11]. The mainstay treatment for Stage IV (Tx Nx M1) colon and rectal cancer is chemotherapy; however, the poor prognosis of Stage IV CRC calls for the development of more targeted treatments; thus, biomarkers are greatly needed to tailor adjuvant therapy and more accurately guide the selection of chemotherapy regimens in CRC patients of all stages.

In this review, we describe four ‘traditional' tumor markers that have been widely studied as reflections of CRC heterogeneity: microsatellite instability (MSI) or DNA mismatch repair (MMR) deficiency, the CpG island methylator phenotype (CIMP), somatic mutations in *BRAF*, and somatic mutations in *KRAS.* The former two attributes (MSI/MMR and CIMP) represent global phenomena across the colorectal tumor genome indicative of genetic dysfunction, whereas the latter two (*BRAF* and *KRAS* mutation status) are point mutations that may act as drivers of CRC development. Here, we briefly review ways in which these tumor markers have been used to better characterize CRC epidemiology and may be used to guide clinical decision-making. Going beyond these traditional tumor markers, we also briefly review some more novel markers that are likely to be of clinical significance. Lastly, recognizing that none of these individual tumor markers are isolated attributes but, rather, a reflection of broader tumor phenotypes, we review some of the hypothesized etiological pathways of CRC development and their associated clinical differences.

## Traditional Tumor Markers in Relation to Etiology, Epidemiology and Treatment

### Microsatellite instability or mismatch repair

Microsatellite instability (MSI) is recognized by the presence of a high frequency of genetic alterations in microsatellite DNA repeat sequences (i.e., increased or decreased numbers of repeats in tumor DNA relative to DNA from normal surrounding tissue), resulting from deactivation of the DNA mismatch repair system. Approximately 15% of colorectal tumors exhibit high levels of MSI (MSI-high) [12]. In the majority of such tumors, MSI is due to epigenetic silencing of a DNA mismatch repair (MMR) gene (e.g. hypermethylation of the *MLH1* promoter), although ∼20% of MSI-high tumors are due to germline mutations in one of the MMR genes (*MLH1,* *MSH2,* *MSH6* or *PMS2*) (i.e. Lynch Syndrome) [13]. Compared with patients with microsatellite stable (MSS) CRC, patients with MSI-high CRC are more likely to be smokers [14–17], more likely to consume alcohol [15, 16], and are less likely to be obese [18].

MSI status is consistently associated with survival of CRC: a recent meta-analysis showed MSI-high CRC to be associated with a 40% better overall survival rate than MSS CRC [12]. Even when matched for stage, individuals with MSI-high CRC—particularly in the proximal colon—appear to have a better prognosis than those with MSS CRC [19]. Emerging data suggest that therapy should be tailored to MSI status in both early-stage and advanced CRC. In particular, MSI has been shown to predict lack of benefit from adjuvant 5-FU in Stage II–III colon cancer (and possible harm in Stage II patients) [20]; however, the value of an MSI as a predictive marker with modern combination chemotherapy regimens—such as FOLFOX and FOLFIRI (5-FU with irinotecan)—is uncertain*.* In a recent trial of Stage III colon cancer treated with adjuvant FOLFOX, MSI status was not predictive of outcomes overall [21]. In retrospective analyses of another adjuvant trial for Stage II–III colon cancer, those with MSI-high tumors again had a better prognosis but there was no association between MSI status and benefit from oxaliplatin [22]. In contrast, MSI-high status has been shown to predict benefit from adjuvant irinotecan (IFL regimen) in at least one trial; however, MSI has not reliably served as a predictor of benefit from combination chemotherapy with 5-FU and irinotecan [23–25]. Observed differences between MSI as marker for 5-FU response *vs.* response to combination chemotherapy may reflect differences across trials in specific chemotherapy regimens and/or variability in multivariate models, as newer models often account for other tumor attributes, such as *KRAS* and *BRAF* mutation status [26]; thus, while MSI status is largely accepted as a prognostic marker, its role as a predictive biomarker with modern combination chemotherapy regimens remains controversial.

### CpG island methylator phenotype

The genome is interspersed with dense CpG-rich regions, termed ‘CpG islands', which are found in the promoter regions of roughly half of all genes [27]. Methylation of these islands often results in gene silencing [27–29], which can be a driver of carcinogenesis (e.g. hypermethylation of the *MLH1* promoter region) [30–32]. Two patterns of CpG island methylation have been observed in CRC: low-level methylation that increases incrementally with age, and high-level methylation of a particular subset of CpG islands, resulting in gene silencing [30]. CRC tumors exhibiting the latter pattern are referred to as ‘CpG island methylator phenotype (CIMP)-positive'. Approximately 30–40% of tumors located in the proximal colon and 5–15% of tumors in the distal colon and rectum can be classified as CIMP-positive [33]. Compared with CRC patients with non-CIMP tumors, those with CIMP-positive CRC are more likely to be smokers [14, 34] and less likely to be obese [35].

Although CIMP is thought to play an important role in the natural history of CRC, studies of the association between CIMP status and survival of CRC have been inconsistent [36, 37]. In part, investigation of CIMP as a prognostic or predictive biomarker has been slowed by the lack of consensus regarding which CIMP panel to use in assaying this attribute. Despite the lack of a ‘gold-standard' CIMP assay, there has been some suggestion of an association between CIMP and a favorable response to 5-FU [38–41]; however, studies assessing the use of CIMP as a predictor for response to FOLFOX therapy have not observed CIMP to be a valuable biomarker [42]; thus, the use of CIMP status as a predictive marker for conventional chemotherapy will require further investigation.

### Somatic mutations in *BRAF* and *KRAS*

Activating mutations in *BRAF* and *KRAS *are evident in approximately 5–15% and 30–45% of colorectal tumors, respectively [36, 43–48]. Such mutations result in cell proliferation and inhibition of apoptosis due to dysregulation of the MAPK signaling pathway. As part of the same pathway, *BRAF *and *KRAS *mutations tend to be mutually exclusive molecular events in CRC development [49]. The *BRAF* V600E mutation accounts for approximately 90% of all *BRAF *mutations observed in CRC [50, 51]. In comparison, mutations in ‘hot-spot' codons 12 and 13 of exon 2 account for approximately 90% of all *KRAS *mutations in CRC [45, 52–54]. Compared with patients with *BRAF*-wildtype tumors, patients with *BRAF*-mutated tumors tend to be diagnosed at a later age [43], are more likely to be female, and are more likely to be smokers [14, 34]. In contrast, few epidemiological differences have been noted by *KRAS *mutation status [55–57].

While there is substantial evidence, from retrospective analyses of cohort and randomized clinical trials, that mutated *BRAF* in CRC is a marker of poor prognosis [26, 58], it is an active question as to whether *BRAF*-mutant patients should receive more aggressive ‘up-front' chemotherapy. One small Phase II trial recently suggested improved survival associated with the use of FOLFOXIRI plus bevacizumab in patients with metastatic *BRAF*-mutated CRC [59]; however, the predictive benefit of *BRAF* has not been shown for either cytotoxic chemotherapy or anti-epidermal growth factor receptor (EGFR) treatment [60, 61]. Notably, the success of *BRAF*-inhibitors seen in the treatment of melanoma has not been paralleled in CRC [62]; thus, *BRAF* is best considered as only a prognostic marker at this time.

In contrast, *KRAS *mutation status is well established as a predictive marker for anti-epidermal growth factor receptor (EGFR) therapy in metastatic CRC. While initial studies of anti-EGFR therapy in metastatic CRC produced mixed results, it was soon shown that efficacy of treatment could be predicted by *KRAS* mutation status [63–65]. Specifically, responses were observed in patients whose tumors did not exhibit *KRAS* exon 2 mutations, while no response—or even harm—was seen in patients with *KRAS*-mutant tumors [66, 67]. Multiple retrospective studies have now shown that *KRAS* testing restricted to exon 2 misses 15–17% of cases resistant to anti-EGFR therapy [68, 69]. ‘Expanded *RAS*' testing is now advocated, which also tests exons 3 (codon 61) and 4 (codons 117 and 146) of *KRAS* and exons 2–4 of *NRAS* [70]. The utility of *KRAS *mutation testing for guiding treatment selection in patients with earlier stage colon cancer has not been supported [71].

## Additional Tumor Markers with Potential Relevance to Clinical Management

### Somatic mutations in PIK3CA

Phosphatidylinositol 3-kinase (PI3K) is a lipid kinase, critical in the initiation of signaling pathways for cell proliferation, migration and survival [72, 73]. Mutations in the gene encoding the catalytic sub-unit of PI3K (i.e. the phosphatidylinositol-4, 5-bisphosphate 3-kinase, catalytic sub-unit alpha [*PIK3CA*] gene) can result in constitutive activation of PI3K signaling and, thus, disregulated cell proliferation contributing to the development of cancer [74]. Somatic *PIK3CA *mutations have been noted in approximately 10–20% of CRCs [73, 75–83]. Studies characterizing patients with *PIK3CA*-mutated CRC have suggested that tumors exhibiting these mutations are more likely to be located in the proximal colon [77, 80, 84], and to exhibit *KRAS *mutations [75–80, 84] than *PIK3CA*-wildtype colorectal tumors.

PI3K is distinct from the RAS/RAF pathway but mutations in *PI3KCA* may also affect responsiveness to anti-EGFR therapy, especially mutations in exon 20 [61]. PI3K pathway inhibitors have been developed, but have largely failed to show benefit in treatment [85, 86]. *PIK3CA* mutations may, however, suggest benefit from aspirin for secondary prevention, since large studies have demonstrated that aspirin reduces adenoma and CRC formation in individuals with *PIK3CA*-mutated primary CRC [87, 88].

### Hypermutation

Recent findings from The Cancer Genome Atlas (TCGA) network's genome-wide analysis of CRC indicated that 16% of colorectal tumors displayed a significantly higher density of somatic sequence mutations than expected (i.e. hypermutated) [89]. The majority of these cases were also MSI-high and/or CIMP-positive, although a previously unrecognized class of hypermutated CRCs was also observed. Further investigation has led to the identification of the polymerase genes *POLE* and *POLD1* which, when mutated in the germline or somatically, can result in this hypermutated phenotype with >1 000 000 base substitutions per tumor [90]. The clinical relevance of mutations in *POLE *and *POLD1* and, more generally, hypermutated status, is emerging; for example, recently published data notes that MSI-high CRCs respond to programmed-death-1 (PD-1) checkpoint inhibitors, while MSS CRCs do not [91]. This finding is believed to reflect the fact that MSI CRCs have a mutation rate that is 20 times higher than that in MSS CRC, and that the neo-antigens resulting from this allow for better efficacy for immune-modulating agents. Further research into how hypermutated CRC responds to available therapies is needed, and the development of therapies targeting hypermutated CRC genomes is warranted.

### Other emerging tumor markers

There continues to be considerable interest in identifying predictive molecular markers for chemotherapy effect, both in the CRC adjuvant and metastatic settings [92]. Candidate biomarkers that have been heavily scrutinized include mutant *TP53*, thymidylate synthase (TS) expression, *MEK*, and amplified ERCC1, among others [93–96]. Unfortunately, none of these markers has been established as a predictive marker that is ready to be used clinically. Other future directions include dual pathway blockade to mediate mechanisms of resistance [97, 98]; however, such an approach has thus far been found to be sub-optimal when applied to the clinical setting, often increasing toxicity but not improving treatment outcomes [99, 100].

## Molecular Classifications of CRC Subtypes Based on Proposed Etiological Pathways

Although much research has been devoted to the epidemiological and clinical implications of the previously-described CRC tumor markers individually, these highly correlated markers may be of even greater utility to clinical research when considered in combination. Previously-described pathways of CRC development have been suggested to result in tumor subtypes that can be distinguished by specific combinations of MSI, CIMP, *BRAF*-mutation, and *KRAS*-mutation status. Preliminary research suggests marked differences in prognosis across these pathway-informed tumor subtypes, suggesting opportunities for more closely targeted clinical management of CRC.

### Traditional adenoma–carcinoma sequence

The majority (55–70%) of colorectal tumors arise via the well-characterized sequential transition from normal mucosa to adenoma to carcinoma [101–103]. This traditional pathway involves an accumulation of activating mutations in oncogenes and deactivating mutations in tumor-suppressor genes, and appears to result in CRC resulting from MSS, non-CIMP, and absent *BRAF *or *KRAS *somatic mutations [101]. Tumors resulting from this pathway are typically also characterized by driver mutations in *APC* and by chromosomal instability (i.e. large genomic alterations including the gain or loss of chromosomal regions and/or aneuploidy) [101, 104]. Given that this pathway is, by far, the predominating pathway responsible for CRC development, studies that have not incorporated information on CRC molecular phenotype are likely to most closely reflect the epidemiology and clinical course of tumors resulting from this pathway. In particular, these tumors tend to be associated with a more favorable prognosis than *BRAF*-mutated CRC, but a less favorable prognosis than MSI-high CRC [105, 106].

### Serrated pathway

An estimated 20–30% of all CRCs develop through a serrated neoplasia pathway, named for the saw-toothed pattern of crypts in the precursor polyps [104, 107]. Precursor lesions in this pathway differ from those reflective of the traditional adenoma–carcinoma sequence, not only in appearance, but also in molecular attributes and in rates and risk of progression [104, 107–109]. Classification schemes for delineating serrated CRC based on molecular attributes continue to evolve; however, serrated CRC is generally distinguished by the presence of CIMP and mutated *BRAF *or *KRAS* [101, 105, 107].

In addition to molecular differences, several differences between serrated and non-serrated CRC have been reported in terms of genetic predisposition, anatomical site, and tumor aggressiveness [110–112]. Several studies suggest that susceptibility loci for CRC identified from genome-wide association studies (GWAS) are associated with early precursors for non-serrated CRC (adenomas), but not with serrated CRC precursors (serrated polyps) [110, 113]. Colorectal tumors exhibiting serrated molecular features are also more likely to present as proximal colon cancers than as distal colon or rectal cancers [4]. Because proximal tumors are more likely to present at later stages [114, 115], this proximal distribution of serrated CRC-defining attributes could translate to a later stage at diagnosis in serrated *vs.* non-serrated CRC cases; however, previous studies have not consistently demonstrated differences in the distribution of stage by *BRAF *mutation [37, 116, 117], CIMP [36, 118], or *KRAS *mutation status [37, 119].

The existence of the serrated pathway has implications for CRC screening programs: e vidence suggests that serrated tumors may develop more rapidly than other types of CRC, as the tumor markers indicative of serrated CRC are more prevalent in cancers arising within 3–5 years of a colonoscopy (i.e. interval cancers) [120, 121]. This probably reflects the more aggressive nature of CRC arising from the serrated pathway, but also probably reflects the fact that serrated polyps are often more difficult to detect using standard screening techniques: they are less likely than adenomas to bleed, making detection by fecal occult blood testing unlikely [122]; since serrated polyps are more likely to develop in the proximal colon [107], they are less likely to be identified through sigmoidoscopy and, because of their sessile, minimally elevated morphology, serrated polyps can also be difficult to detect via colonoscopy [104]. If colonoscopy and other screening methods are less efficacious for the prevention and early detection of serrated CRC than for other forms of CRC, this shortcoming will present a considerable public health challenge.

In a recent analysis of CRC subtype-specific survival, colorectal tumors exhibiting a serrated phenotype marked by mutated *BRAF*, CIMP-positive, and MSS status, were associated with the poorest prognosis [105]; specifically, patients with these serrated cancers were more than twice as likely to die from their disease than patients with tumors exhibiting a phenotype indicative of the traditional adenoma–carcinoma sequence. In contrast however, patients with CRC exhibiting mutated *BRAF*, CIMP-positive, and MSI-high were significantly less likely to die from their disease than those with traditional adenoma–carcinoma pathway tumors [105]; thus, even among patients with CRC suggestive of serrated pathway origins, there is considerable heterogeneity in clinical outlook. This supports the need to consider multi-marker tumor phenotypes in projecting CRC prognosis and in guiding clinical management of disease.

### Alternative pathway

Although sometimes grouped with serrated pathway cancers, colorectal tumors that are *KRAS*-mutated, CIMP-low, and MSS have been suggested to arise from an ‘alternative pathway’ [101, 104, 107, 123, 124]. The low levels of CIMP methylation seen in this group could represent a second type of CIMP [48], or may reflect a high level of methylation at different loci than those measured on current CIMP panels [104]. The finding that silencing of the DNA repair gene *MGMT *by promoter hypermethylation is associated with *KRAS-*mutated and CIMP-low status [124–126] suggest that *MGMT *methylation may be another characteristic of this alternative pathway. It is unclear which precursor lesions might be indicative of this pathway, although possibilities include traditional serrated adenomas, tubulovillous adenomas with serrated features, and serrated polyps with dysplasia [101, 107]. Although previous studies have not consistently found *KRAS *mutation and CIMP status individually to be significant indicators of prognosis, at least one recent study has found that CRC with a *KRAS*-mutated/CIMP-low phenotype indicative of this alternative pathway confer a significantly poorer prognosis than tumors derived from the traditional adenoma–carcinoma sequence [105, 106].

## Conclusions and Future Directions

With the exception of *KRAS *mutation testing for Stage IV CRC, current clinical practice for the management of CRC does not involve an assessment of a tumor’s molecular phenotype; however, recognizing that CRC is a heterogeneous disease, there are great opportunities to improve CRC prognosis by better incorporating information on tumor biology into treatment decisions and the design of targeted treatment strategies. Even in the absence of agents specifically targeting the treatment of CIMP-positive or *BRAF*-mutated or MSI-high CRC, these markers—alone and particularly in combination—provide insights into the natural history of CRC. In some instances, these markers may also serve as prognostic or predictive markers, providing even greater incentive to collect this information in clinical settings.

As we evolve a better understanding of the diverse pathways that lead to CRC and improve our recognition of the driver mutations and molecular events that contribute to those pathways, approaches to the clinical management of CRC will also need to evolve and improve. Aggressive serrated *BRAF*-mutated/CIMP-positive/MSS tumors will probably necessitate more aggressive treatment and, potentially, different treatment agents than CRC exhibiting high levels of MSI or CRC resulting from the traditional adenoma–carcinoma sequence. As we continue to gain insight into the heterogeneity of CRC biology, etiology, epidemiology and clinical profile, the clinical management of CRC will continue to evolve in order to incorporate this information into clinical decision-making, to personalize and improve the care of CRC patients.

Funding: This work was supported by the National Cancer Institute, National Institutes of Health (K07CA172298 to A.I.P., T32CA09168 to J.M.K.), the National Center for Advancing Translational Health Sciences, National Institutes of Health (KL2TR000421 to J.M.K.), and a Young Investigator Award from the Conquer Cancer Foundation of the American Society for Clinical Oncology (to S.S.). The content is solely the responsibility of the authors and does not necessarily represent the official views of the National Institutes of Health.

*Conflict of interest statement:* none declared.
